# Energy Sensing versus 2-Oxoglutarate Dependent ATPase Switch in the Control of *Synechococcus* P_II_ Interaction with Its Targets NAGK and PipX

**DOI:** 10.1371/journal.pone.0137114

**Published:** 2015-08-28

**Authors:** Jan Lüddecke, Karl Forchhammer

**Affiliations:** Interfaculty Institute for Microbiology and Infection Medicine, Division Organismic Interactions, University of Tübingen, Auf der Morgenstelle 28, D-72076, Tübingen, Germany; Centre National de la Recherche Scientifique, Aix-Marseille Université, FRANCE

## Abstract

P_II_ proteins constitute a superfamily of highly conserved signaling devices, common in all domains of life. Through binding of the metabolites ATP, ADP and 2-oxoglutarate (2-OG), they undergo conformational changes which allow them to regulate a variety of target proteins including enzymes, transport proteins and transcription factors. But, in reverse, these target proteins also modulate the metabolite sensing properties of P_II_, as has been recently shown. We used this effect to refine our P_II_ based Förster resonance energy transfer (FRET) sensor and amplify its sensitivity towards ADP. With this enhanced sensor setup we addressed the question whether the P_II_ protein from the model organism *Synechococcus elongatus* autonomously switches into the ADP conformation through ATPase activity as proposed in a recently published model. The present study disproves ATPase activity as a relevant mechanism for the transition of P_II_ into the ADP state. In the absence of 2-OG, only the ATP/ADP ratio and concentration of ADP directs the competitive interaction of P_II_ with two targets, one of which preferentially binds P_II_ in the ATP-state, the other in the ADP-state.

## Introduction

P_II_ signaling proteins are at the core of metabolic regulation in almost all free-living bacteria, in nitrogen-fixing archaea and in the chloroplasts of red algae and plantae, thereby constituting one of the largest signaling protein families in nature [1–6]. They regulate anabolic reactions related to nitrogen metabolism and do so at all levels of regulation: from transport of nitrogenous compounds to nitrogen-dependent gene expression. P_II_ proteins perceive metabolic information by competitive binding of ATP or ADP and by synergistic binding of ATP and 2-OG [7–11]. This core sensing function of P_II_ proteins appears to be highly conserved across the domains of life, as revealed by analyzing P_II_ proteins from bacteria, archaea and plants [1–6, 12]. P_II_ proteins operate on the basis of a highly conserved structure: they are homo-trimers composed of subunits which in bacteria are almost invariantly 112 amino acids long. A typical P_II_ trimer consists of a highly conserved core structure from which three large flexible loops (one per subunit) extend into the solvent. The basal part (proximal to the body of the protein) of these so-called T-loops takes part in effector molecule binding, whereas the apical region typically mediates the interaction with diverse receptors. Two smaller loops projecting into the inter-subunit cleft from opposing subunits, the B- and C-loops, also take part in effector molecule binding [3, 6, 13]. The adenylate nucleotides ATP and ADP compete for this binding site. In the ATP bound conformation, cyanobacterial P_II_ readily interacts with the controlling enzyme of the arginine synthesis pathway, N-acetyl-L-glutamate kinase (NAGK). ADP occupies the same site as ATP but engages a distinct arrangement of interactions, thereby favoring a characteristic vertically extended orientation of the T-loop [13, 14]. In this conformation, the P_II_ protein GlnK from *E*. *coli* interacts with the ammonium channel AmtB [15] and the *Synechococcus* P_II_ protein interacts with PipX, a co-activator of the transcription factor NtcA [16]. When occupied with Mg^2+^-ATP, the three sites can also bind 2-OG, whereas ADP does not support the binding of 2-OG [17, 18]. Binding of 2-OG leads to a unique folding of the T-loop, which prevents various T-loop-dependent P_II_ interactions [3, 8].

Recently, the effects of various ATP/ADP ratios on the interaction of the cyanobacterial P_II_ protein from *Synechococcus elongatus* with its targets NAGK and PipX were investigated. Whereas the interaction of P_II_ with NAGK was only very weakly affected by varying ATP/ADP ratios, the interaction with PipX was highly sensitive towards increasing ADP levels [13]. NAGK favors the interaction with P_II_ in the ATP-state. By contrast, the interaction of P_II_ with PipX is stimulated by the ADP-state [16]. Complex formation with PipX increases the affinity of P_II_ for ADP, which further enhances the sensitivity towards ADP.

A different mechanism for the role of ATP or ADP and the involvement of 2-OG in P_II_ signaling was proposed for the highly conserved interaction of the *E*. *coli* GlnK with AmtB [19]. In the absence of 2-OG, GlnK slowly hydrolyzed bound ATP, and switched into the ADP-P_II_ complex with the vertically extended T-loops. This change in the T-loop structure allows the interaction of GlnK with AmtB and was proposed to be a general mechanism of signal transduction by P_II_ proteins [19]. However, in order to be able to measure ATP hydrolysis to ADP, 100–150 μM P_II_ protein had to be incubated with 3 μM ATP for 30 min, raising the question of the physiological significance of such a minute activity.

The present work aims to clarify whether P_II_-target interaction is driven by the ATPase switch mechanism or via allosteric metabolite level sensing in a case study for the interaction of *S*. *elongatus* P_II_ with its two known targets, NAGK and PipX. Since these P_II_ targets have opposing preferences for the ATP or ADP-conformation of P_II_, they are ideally suited to monitor the respective P_II_ conformations.

## Results and Discussion

In enzyme assays for NAGK activity, where ATP hydrolysis is coupled to the oxidation of NADH via pyruvate kinase and lactate dehydrogenase, we never observed ATP hydrolysis activity of P_II_ proteins [20]. Possibly the ATPase activity is too low to be detectable by this assay. So we asked the question whether ATP hydrolysis plays any role for *Synechococcus* P_II_ function. The ATPase switch model proposes that incubation of P_II_ with ATP in the absence of 2-OG switches the P_II_ protein from an ATP-state into an ADP-state and the interaction of P_II_ with its receptors would respond accordingly: P_II_ interactions would switch from P_II_-receptors recognizing the ATP-state to P_II_-receptors recognizing the ADP-state of P_II_. In the case of *Synechococcus* P_II_, the receptor NAGK binds preferentially to the ATP-state of P_II_ [21] whereas the receptor PipX prefers the ADP-state of P_II_ [13, 16]. Therefore, upon a switch from the ATP to the ADP state, P_II_ should change from the NAGK-bound state to the PipX bound state.

**Fig 1:**
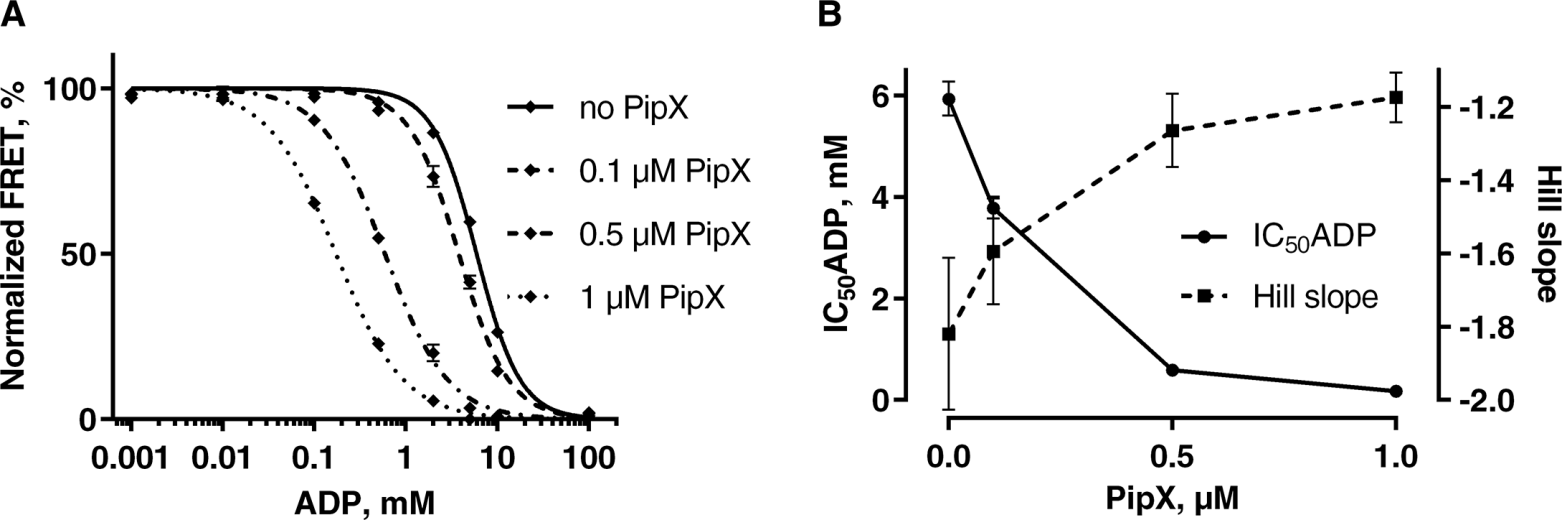
Response of the P_II_-V NAGK-C FRET sensor towards ADP in the presence of different PipX concentrations. (A) At a constant ATP concentration of 1 mM the effect of different ADP concentrations on the P_II_-V NAGK-C FRET was measured in the absence and presence of different PipX concentrations. P_II_-V and NAGK-C were used in concentrations of 0.1 μM. For practical reasons, the data points representing minimum and maximum values of ADP/ATP ratios where in fact derived from measurements without ADP (using the data point at 0.001 mM ADP) or 10 mM ADP without ATP (using the data point 100 mM ADP). Possible trace amounts of contaminating ADP in the ATP solution were not considered here. Mean values of 3 measurements with standard deviation are shown and a sigmoidal dose response curve was fitted using GraphPad Prism 6. (B) The Hill slope and IC_50_(ADP) values derived from the fitted curves of Fig 1A are presented with error bars indicating the 95% confidence intervals.

To test the applicability of the ATPase-switch model for *S*. *elongatus* P_II_-receptor interactions, we employed the recently established P_II_-NAGK FRET sensor, which measures the interaction of P_II_ and its partner NAGK in a highly sensitive manner, exceeding the sensitivity of previously used assays [21]. When P_II_, with its C-terminus fused to the fluorescent Venus protein, interacts with NAGK, with the fluorescent protein Cerulean fused to its C-terminus, the fluorescent proteins come in close proximity resulting in FRET that can be measured as a shift in fluorescence. Despite the size of the attached fluorescence proteins, we previously showed that P_II_-Venus (P_II_-V) and NAGK-Cerulean (NAGK-C) formed functional oligomers and interacted like wild type proteins. Furthermore, the enzymatic activity of hybrid NAGK was comparable to the natural protein [21]. When P_II_-V and NAGK-C are incubated at a fixed ATP concentration of 1 mM and challenged with increasing concentrations of ADP, it turns out that an ADP:ATP ratio of 6:1 is needed to trigger a P_II_-V NAGK-C dissociation which leads to a 50% drop in the FRET signal (Fig 1A, solid line, Fig 1B IC_50_(ADP)). This demonstrates that the P_II_-NAGK interaction is only weakly affected by fluctuating ATP/ADP levels and only under unphysiologically excessive ADP concentrations P_II_-NAGK interaction is impaired [21], which is in accordance with the 2–3 fold higher affinity of P_II_ for ATP than for ADP [22].

**Fig 2:**
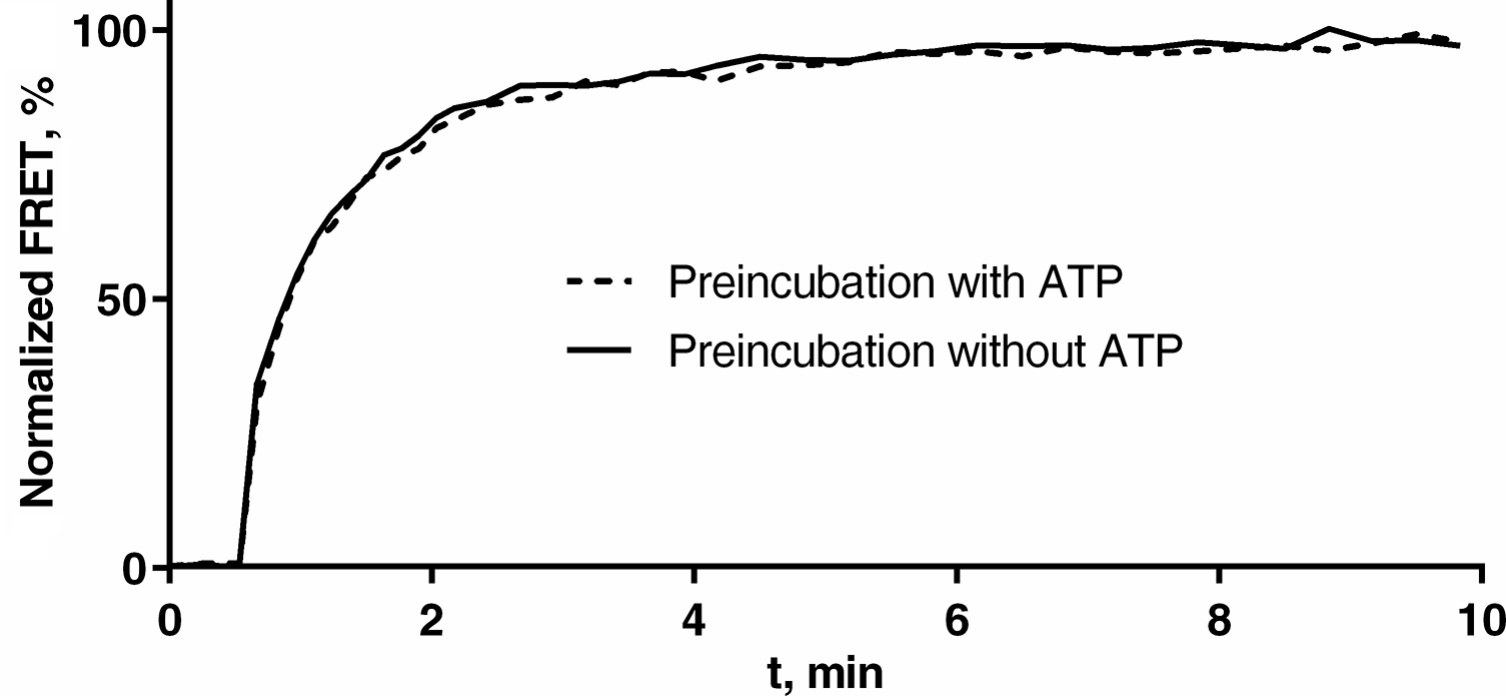
Association of P_II_-V and NAGK-C after P_II_-V preincubation with and without ATP, measured by FRET. P_II_-V (0.1 μM) was either preincubated with 10 μM ATP (solid line) or without ATP (dashed line) for 30 min at 37°C. The FRET measurement was started and after 0.5 min NAGK-C (0.1 μM) was added. In the control experiment ATP (10 μM) was added together with NAGK-C.

We next tested how the system responds to pre-incubation of P_II_ with ATP. According to the ATPase switch model, these conditions should convert P_II_ into the ADP-state and thus, tune down the interaction of P_II_ with ATP-state dependent P_II_ receptors like NAGK. Therefore, we preincubated 0.1 μM P_II_-V with 10 μM ATP at 37°C for 30 min, then added NAGK-C and measured the association of P_II_-V and NAGK-C via FRET. In a control experiment we incubated P_II_-V without ATP, which was added together with NAGK-C. As presented in Fig 2 both setups resulted in a highly similar association curve.

This experiment showed that the proposed ATPase-switch model is not relevant for pure P_II_-NAGK interaction, which was expected, since P_II_-NAGK interaction is not affected by various ADP concentrations as long as ATP is present. However, according to the ATPase switch model, the situation should change in the presence of PipX. Here, the affinity of P_II_ for ADP increases [13], and thus, PipX could stabilize the ADP-state of P_II_ after it arises during the presumptive ATPase cycle. Therefore, we modified the FRET-based P_II_-NAGK interaction assay by adding different amounts of PipX to the sensor protein mixture. FRET was measured in the presence of a fixed concentration of ATP together with increasing concentrations of ADP. If PipX sequestered P_II_ in the ADP-bound state, the interaction between P_II_ and NAGK would be antagonized, since binding of P_II_ to NAGK or PipX is mutually exclusive [16]. As shown in Fig 1A and 1B, this ADP-promoted antagonism by PipX can indeed be seen: in the presence of PipX (dashed lines) the response towards ADP increases, resulting in lowered IC_50_(ADP) values and shallower Hill slopes. Using equimolar concentrations of P_II_-V, NAGK-C and PipX the effect is small, lowering the IC_50_ value from 5.9 to 3.8 mM and changing the Hill slope from -1.8 to -1.6. However, at a tenfold excess of PipX over P_II_-V and NAGK-C, the IC_50_ value of ADP was as low as 0.17 mM. This means that under these conditions an ADP:ATP ratio of 0.17:1 leads to a FRET drop of 50%, representing a 35-fold increase in sensitivity of the sensor towards ADP as compared to the absence of PipX. Thus, a high excess of PipX is able to strongly stabilize the ADP conformation of P_II_. At a 1 μM PipX concentration also the Hill slope is 35% shallower than without PipX. This is advantageous for a sensor as it represents a wider dynamic range. In Fig 1B the IC_50_(ADP) and the corresponding Hill slope were plotted against the concentration of PipX, showing almost saturation at 1 μM of PipX. These data reveal that at high ATP/ADP ratios, NAGK has higher affinity for P_II_ than PipX, whereas with increasing ADP levels, PipX increasingly successfully competes with NAGK for P_II_ binding. The IC_50_(ADP) value indicates the ADP concentration, where NAGK and PipX have the same affinity to P_II_. In principle a similar result, albeit only qualitatively and not quantitatively, was obtained previously by a competition experiment using surface plasmon resonance spectroscopy with immobilized NAGK and applying P_II_ or P_II_-PipX mixtures as analyte [13].

By using a fluorescence platereader for the measurements shown in Fig 1 we were able to follow the time course of the FRET signal after addition of the nucleotides. As shown in S1 Fig, increasing amounts of PipX not only enhance the sensitivity towards ADP but also lead to slower development of a steady FRET level. Even at very low ADP concentrations, a great excess of PipX is able to temporarily occupy P_II_ and block the interaction between P_II_ and NAGK, but ultimately, after an incubation time above one hour, the FRET signal reaches the same level as without PipX (S1D Fig). As an explanation for this interesting observation, we assume that after adding the mixture of excess PipX over NAGK to P_II_ proteins, P_II_ is transiently trapped by the excess of PipX molecules. But due to the higher affinity of NAGK to P_II_, the non-occupied NAGK molecules gain back P_II_ form P_II_-PipX complexes in a slow process that can be visualized in real-time by following the FRET signal.

**Fig 3:**
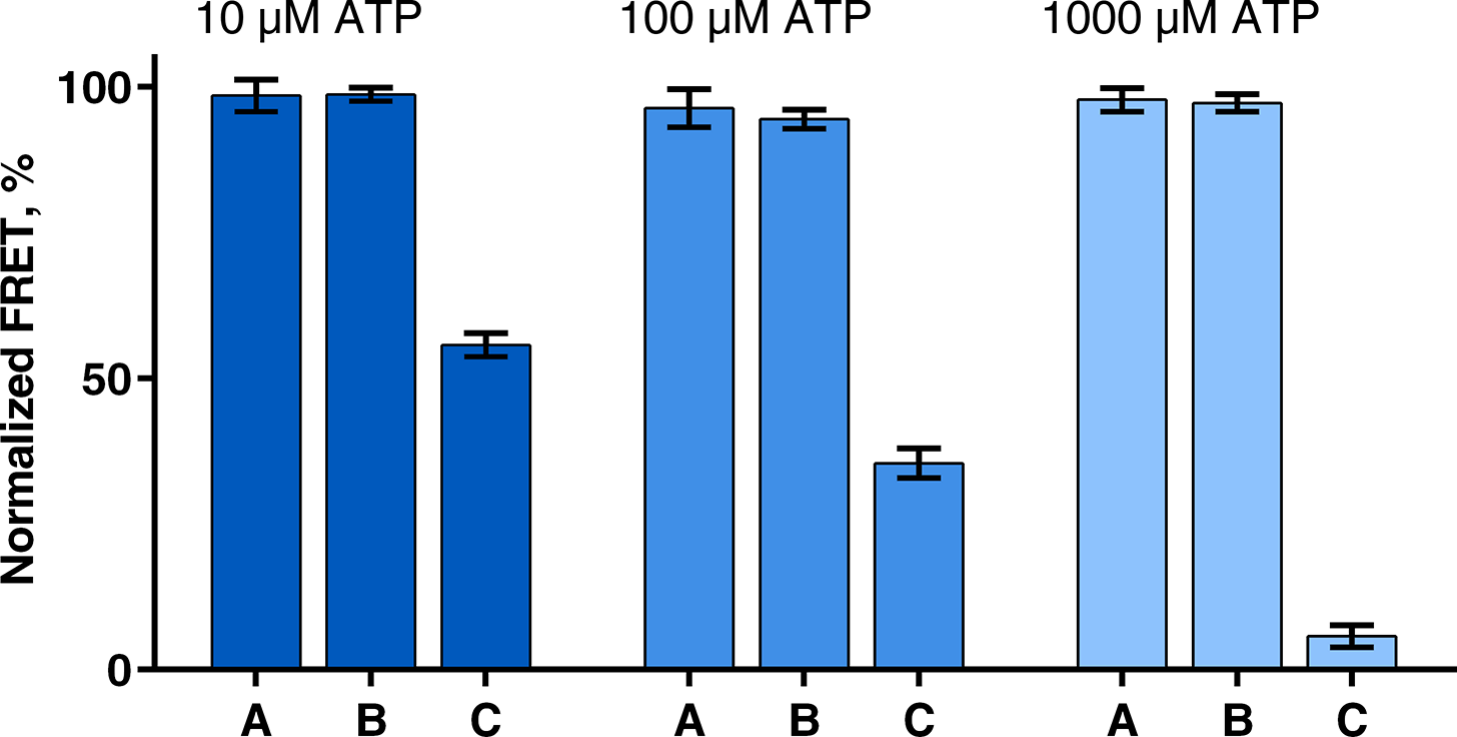
Measuring the ADP production by P_II_. **A:** P_II_-V was incubated with ATP for 1 h at 37°C, then NAGK-C and PipX were added and FRET was measured after 15 min of additional incubation. **B:** P_II_-V was incubated without ATP for 1 h at 37°C, then ATP, NAGK-C and PipX were added and FRET was measured after 15 min of additional incubation. **C:** P_II_-V was incubated without ATP for 1 h at 37°C, then a mixture of ATP, ADP, NAGK-C and PipX was added and FRET was measured after 15 min of additional incubation. Used concentrations: P_II_-V, NAGK-C: 0.1 μM, PipX: 1 μM; ATP (A, B): 10 μM (dark blue), 100 μM (blue), 1000 μM (light blue); ATP+ADP mixture (C): 5 μM each (dark blue), 50 μM each (blue) 500 μM each (light blue). Mean values of 3 experiments with standard deviation are shown.

With this carefully calibrated FRET sensor, already minute quantities of ADP quench P_II_-NAGK interaction. Therefore, this test system precisely reveals the ADP-promoted interaction between P_II_ and PipX and is sensitive enough to resolve any P_II_ partner swapping that may be caused by the postulated ATPase switch mechanism. With this PipX-supplemented P_II_-NAGK FRET setup we finally tested whether an ATPase switch mechanism might be relevant under conditions, where P_II_ can competitively interact with either PipX or NAGK. To maximally increase the sensitivity towards minute amounts of ADP, which may be produced by any ATPase activity of P_II_, we increased the pre-incubation time to 1 h at 37°C and tested ATP concentrations of 1000, 100 and 10 μM with 0.1 μM of P_II_-V. After the pre-incubation, during which the ATPase activity may switch the conformation of P_II_, 0.1 μM of NAGK-C and 1 μM of PipX were added and the mixture was further incubated for 15 minutes, to give P_II_-V and NAGK-C time to form a complex, before the FRET signal was measured. As negative control, P_II_-V was pre-incubated for 1 h without ATP and the nucleotide was added together with NAGK-C and PipX in the final mixture. In a positive control, P_II_-V was pre-incubated without ATP and a 1:1 mixture of ATP and ADP was added together with NAGK-C and PipX, to simulate that 50% of ATP had been converted to ADP and to reveal how ADP, which might be formed during the pre-incubation period, affects the distribution of P_II_ between NAGK and PipX. These controls equal situations where each ATP binding site of P_II_ would have converted approximately 0.8, 8 or 80 molecules of ATP (for 10, 100 or 1000 μM ATP concentration, respectively) per minute during the 1 h pre-incubation period. As shown in Fig 3, there is clearly no difference between the experiments where P_II_ was pre-incubated with ATP and the negative controls without ATP during pre-incubation. By contrast, the positive control (addition of ADP) shows a 44, 75 and 94% lower FRET signal (for the 5, 50 and 500 μM ATP and ADP mixtures, respectively), which also shows that not only the ATP/ADP ratio but also the absolute concentration of ADP is important for the interaction between P_II_ and PipX.

These experiments clearly demonstrate that an ATPase switch controlled regulatory mechanism is not relevant for *S*. *elongatus* P_II_ receptor interactions and that P_II_ does not possess relevant ATPase activity. The sole factors that influence the distribution of P_II_ between its targets PipX and NAGK *in vitro* are the ratios as well as absolute concentrations of ATP, ADP and 2-OG. Whereas the interaction of P_II_ with NAGK is not sensitive towards moderate ADP concentrations, PipX interaction is tuned by physiologically relevant ADP concentrations (in detail shown by Zeth et al. [13]).

The present study does not rule out that the ATPase switch model applies for the interaction between the GlnK and AmtB. However, it points to the necessity to characterize the response of P_II_ signaling towards the metabolite effectors 2-OG, ATP and ADP in each different type of interaction. Due to the enormous complexity of the P_II_ signal transduction mechanism, little differences at the P_II_ structural level can have considerable functional consequences.

## Materials and Methods

### Cloning and protein purification

Cloning and affinity purification of the fusion proteins P_II_-V and NAGK-C was described earlier [21]. In short, P_II_-V was purified from the GlnB deficient *E*. *coli* strain RB9060 [23] using the Strep-Tag approach [24]. NAGK-C and PipX were purified from *E*. *coli* BL21 cells using a 6xHis-Tag [25, 26]. The proteins were stored in a buffer containing 50 mM Tris-HCl pH 7.8, 100 mM KCl, 5 mM MgCl_2_, 0.5 mM EDTA, 1 mM DTT and 50% glycerol at -20°C.

### FRET measurements and corrections

Fluorescence measurements were performed either using a FluoroMax‑2 (HORIBA Europe GmbH, Berlin, Ger) in 1 ml cuvettes (P_II_-V NAGK-C association kinetic, measuring of ADP production) or a Tecan Infinite 200 platereader (Tecan Group Ltd., Männedorf, Switzerland) in 96-well plates (measurements of the impact of ADP on the FRET sensor in the presence of varying PipX concentrations). The reaction buffer was the same in all cases and contained 50 mM imidazole (pH 7.5), 50 mM KCl, 20 mM MgCl_2_, 0.5 mM DTT and varying concentrations of ATP and ADP.

For the FluoroMax-2 experiments the excitation/emission wavelengths were set to 433/475 nm for donor (Cerulean) measurements, 515/525 nm for direct acceptor (Venus) measurements and 433/525 nm for FRET measurements. The bandwidth was set to 1 nm. For the association kinetics the FRET signal was measured every 8 s, with the shutter closed between measurements to prevent bleaching.

The Tecan Infinite 200 measurements were done in bottom reading mode using a volume of 250 μL and excitation/emission wavelengths of 430/475 nm for donor (Cerulean) measurements, 430/535 nm for FRET measurements and 495/535 nm for direct acceptor (Venus) measurements. The excitation/emission bandwidth was fixed at 9/20 nm, respectively. The number of flashes was set to 30, the integration time was 100 μs and the settle time was set to 20 ms. The gain was adjusted manually in control experiments to achieve measurement values between 30,000 and 40,000. Blank measurements, containing just the buffer, were subtracted from sample measurements during the evaluation.

The *N*_FRET_ formula developed by Xia and Liu [27] was used to subtract the direct Venus excitation and bleedthrough from Cerulean into the Venus channel from the FRET signal, using correction factors, and normalize it to donor and acceptor concentrations. The correction factors *a* and *b* were determined in separate measurements containing only P_II_-V or NAGK-C.

## Supporting Information

S1 Fig Response of the P_II_-V NAGK-C FRET sensor towards ADP in the presence of different PipX concentrations at different time points. At a constant ATP concentration of 1 mM the effect of different ADP concentrations on the P_II_-V NAGK-C FRET was measured in the absence of PipX (A) or in the presence of 0.1 μM PipX (B), 0.5 μM PipX or 1.0 μM PipX. P_II_-V and NAGK-C were used in concentrations of 0.1 μM. The ATP/ADP mixtures were pipetted onto a 96-well plate, the mixtures of P_II_-V, NAGK-C and PipX were preincubated in the reaction buffer at 37°C for 20 min, added to the plate and FRET was measured every 15 min. For practical reasons, the data points representing minimum and maximum values of ADP/ATP ratios where in fact derived from measurements without ADP (using the data point at 0.001 mM) or 10 mM ADP without ATP (using the data point 100 mM ADP). Mean values of 3 measurements are shown and connected by lines for better readability.
(TIF)
