# Supplementary figures and images for: Energy Sensing versus 2-Oxoglutarate Dependent ATPase Switch in the Control of Synechococcus PII Interaction with Its Targets NAGK and PipX
Source: PLoS One. 2015 Aug 28;10(8):e0137114. doi: 10.1371/journal.pone.0137114 (PMC4552645; doi:10.1371/journal.pone.0137114)

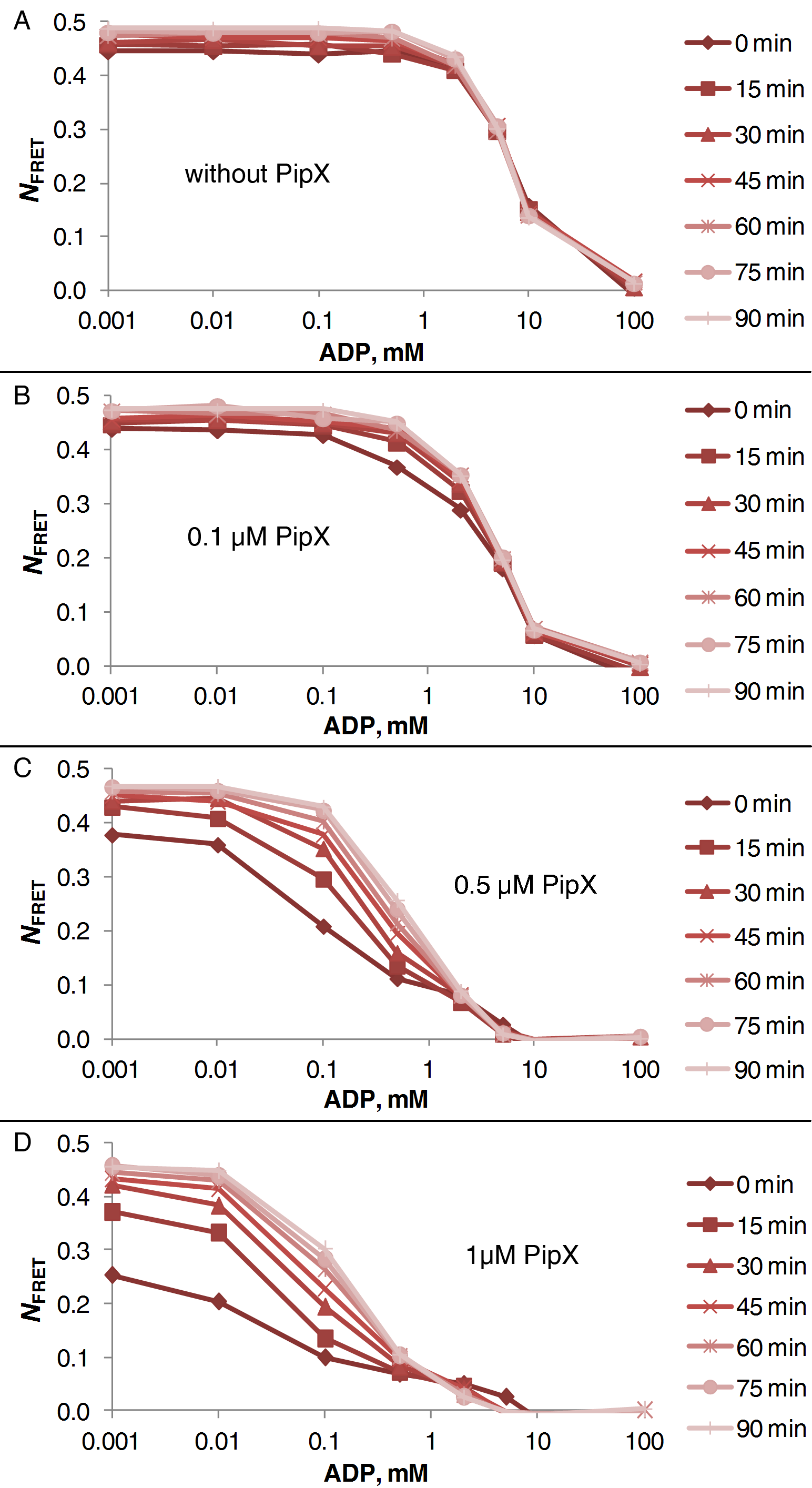

Supplement: S1 Fig — At a constant ATP concentration of 1 mM the effect of different ADP concentrations on the PII-V NAGK-C FRET was measured in the absence of PipX (A) or in the presence of 0.1 μM PipX (B), 0.5 μM PipX or 1.0 μM PipX. PII-V and NAGK-C were used in concentrations of 0.1 μM. The ATP/ADP mixtures were pipetted onto a 96-well plate, the mixtures of PII-V, NAGK-C and PipX were preincubated in the reaction buffer at 37°C for 20 min, added to the plate and FRET was measured every 15 min. For practical reasons, the data points representing minimum and maximum values of ADP/ATP ratios where in fact derived from measurements without ADP (using the data point at 0.001 mM) or 10 mM ADP without ATP (using the data point 100 mM ADP). Mean values of 3 measurements are shown and connected by lines for better readability. (TIF) [file pone.0137114.s001.tif]
